# Designing Integrated Virtual Care Partnerships: Insights from a Practice-Based Case Series at Mayo Clinic

**DOI:** 10.1016/j.mcpdig.2026.100377

**Published:** 2026-06-01

**Authors:** Lindsey M. Philpot, Margaret A. McIntee, Elton A. Mosman, John M. Davis, Megan Dulohery Scrodin, Brian M. Dougan, Stephanie L. Hansel, Xiao Jing Wang, Ziad Zoghby, Jon O. Ebbert

**Affiliations:** a Department of Medicine, Community Internal Medicine, Geriatrics, and Palliative Care, Mayo Clinic, Rochester, MN; b Department of Quantitative Health Sciences, Epidemiology, Mayo Clinic, Rochester, MN; c Department of Medicine, Administrative Services, Mayo Clinic, Rochester, MN; d Department of Medicine, Rheumatology, Mayo Clinic, Rochester, MN; e Pulmonary, Critical Care, and Sleep Medicine, Mayo Clinic, Rochester, MN; f Department of Medicine, General Internal Medicine, Mayo Clinic, Rochester, MN; g Department of Medicine, Gastroenterology and Hepatology, Mayo Clinic, Rochester, MN; h Department of Medicine, Nephrology and Hypertension, Mayo Clinic, Rochester, MN

## Abstract

Access to high-quality primary and specialty care remains a persistent challenge in the United States, with millions of individuals facing barriers due to geography, provider shortages, and health inequities. As health care systems seek innovative solutions, virtual care providers have emerged as critical partners in expanding access, enhancing continuity, and supporting transitions across the health care continuum. This manuscript explores the integration of virtual care providers within an academic medical center, highlighting the dual roles of collaborative and referral models in bridging gaps between traditional outpatient care and virtual health services. Drawing on practical experience, we identify core design principles (acute access, continuity, convenience, cost, reach, and transitions) that underpin successful partnerships and sustainable adoption. These insights underscore the importance of aligning virtual care provider relationships with existing organizational culture, secure data sharing, and supportive reimbursement structures. As academic medical centers and health systems nationwide navigate the evolving landscape of virtual health, thoughtful integration of virtual care providers offers a pathway to address disparities, improve patient outcomes, and advance the quality and reach of care delivery.

Access to timely, high-quality health care remains a persistent challenge in the United States, with nearly 100 million people in the United States lacking access to primary care,^1^ and over one-quarter of Americans facing barriers to specialty care annually.^2^ Limited access to primary care contributes to an estimated 60,000 preventable deaths each year,^3^ whereas insufficient specialty care is linked to increased hospitalization and mortality among vulnerable populations.^4^ Disparities are further exacerbated by factors such as sex, race, geography, and rurality, resulting in an annual economic burden of $320 billion due to health inequities.^5^

In response to these challenges, health care systems are increasingly looking to virtual provider care models,^6^ in which licensed medical professionals deliver care remotely using secure, Health Insurance Portability and Accountability Act-compliant software. The US Department of Health and Human Services defines virtual care providers as clinicians who deliver care, monitor outcomes, and document encounters within telehealth platforms.^7^ To date, published studies have described the experiences of virtual care providers supporting behavioral and mental health,^8^^,^^9^ urgent^10^ and primary care services,^11^^,^^12^ and for condition-specific care (eg, irritable bowel syndrome,^13^ and insomnia^14^). Although previous studies have examined the effectiveness of virtual care providers in behavioral health, urgent care, and condition-specific interventions, few have explored how integrated medical practices view these providers as extensions of the patient care journey. We conducted an exploratory, practice-based thematic synthesis of 10 practice-based cases of virtual care provider integration within an academic medical center, aiming to elucidate their roles as collaborative care and referral partners, and to derive design principles for successful integration into traditional outpatient care models.

## Methods

### Research Questions and Setting

We applied thematic synthesis to address the following study questions: (1) What roles do virtual care providers play in the care of patients within our traditional, outpatient health care delivery system? and (2) what design principles facilitate the integration of virtual care delivery partners into our traditional, outpatient care delivery system? Virtual care providers were identified based on US Department of Health and Human Services definition: employ team-based care models led by licensed clinicians, utilize medical record systems for clinical documentation, and monitor for patient outcomes. To answer these questions, we reviewed all cases of virtual care provider groups integrated into clinical models across the Department of Medicine at Mayo Clinic in Rochester, Minnesota. Mayo Clinic in Rochester is a tertiary medical center that serves over 1 million unique patients each year. The Department of Medicine is the largest clinical department at Mayo Clinic, with expertise in consultative, primary care, and hospital internal medicine, as well as several specialty and subspecialty areas. Included cases are at varying degrees of integration, from no integration and contract cancellation to full integration.

### Approach

Thematic synthesis is a qualitative approach used to answer research questions across multiple published studies of qualitative findings, like the role of systematic reviews for quantitative observational and controlled studies.^15^ Thematic synthesis advances meta-ethnography and grounded theory methodologies combined with the intent to address practical questions about the need, appropriateness, and acceptance of an intervention.^16^ According to the Cochrane Review, thematic synthesis can be used to synthesize findings across individual qualitative studies or practice-based case studies.^17^ In the present study, we use practice-based case studies of all virtual care partnerships established from July 2022 to July 2025 across the Department of Medicine. In this context, partnerships refer to an arrangement in which Mayo Clinic staff members work with virtual care providers under a referral or a collaborative care contract, enabling them to refer Mayo Clinic patients to the virtual care provider. We leveraged the model presented by Ebbert et al^6^ to ground our work, and to provide definitions for referral and collaborative care models.

Three stages of thematic synthesis were performed for this investigation. First, primary data were collected through one-on-one and group-based interviews with practice members of each virtual care provider partnership and coded line-by-line following the principles of interpretive latent content. At minimum, we interviewed 3 leaders from each practice-based case using semistructured interviews: the initiating physician, the physician practice lead for each practice, and the administrative lead for the partnership. In addition, 6 of the 10 cases requested interviews with the nurse manager or nurse administrator, and 2 cases included specializing psychologists.

Transcripts were used from individual and group-based interviews for coding. To promote coding reliability and analytic rigor, a shared codebook was developed and iteratively refined through team-based discussion. An initial subset of transcripts was independently coded by 2 investigators; coding discrepancies were reconciled through consensus, and code definitions were clarified before coding the remaining transcripts. Throughout analysis, the research team met regularly to review coding decisions and confirm that emergent themes were grounded in the data.

Individual codes were aggregated as patterns emerged and themes were identified in response to our stated research questions. Identified patterns and themes were summarized and presented back to each practice area for refinement and iteration. Analytic themes were developed based on these identified and refined patterns to provide understanding about why the identified themes presented themselves within the data, as well as any limitations inherent within our sample. To be considered an analytic theme within this study, the theme must represent a higher-level concept of detailed transcript data, be recurrent across our individual cases, offer explanatory or interpretive depth from our experiences with virtual care provider partnerships, and be coherent within our study questions.

## Results

### What Roles do Virtual Care Providers Play in the Delivery of Care in Academic Medical Settings?

A synthesis of 10 practice-based cases was conducted to characterize the roles played by virtual care providers within an academic medical center. The included clinical cases existed within primary care settings, specialty medical care settings, and as mental and behavioral health support options for patients from both settings. The Figure summarizes the identified roles of virtual care providers across these domains, indicating whether the care model was structured as collaborative care (i.e., integrated with existing practices) or as a referral-based model. Table 1 provides an overview of each practice-based case, including which practice conceptualized the clinical need (originating practice), which type of virtual care provider was selected, the model type (collaborative, referral, or both), and the design principles derived within each case.

**Figure:**
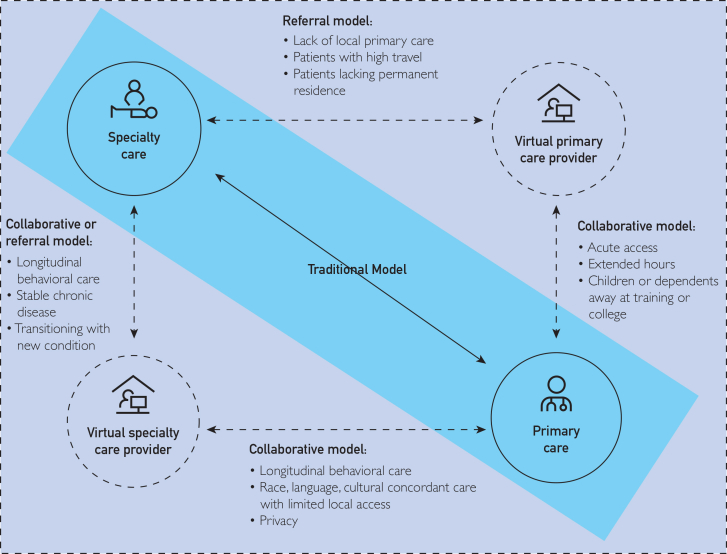
Service delivery model extending traditional medical care to include virtual care providers, derived from Ebbert et al.^6^

**Table 1:** Overview of Models Included Within Thematic Analysis, Including Academic Medical Center Originating Practice, Originating Practice Needs, Virtual Care Provider Type, Model Type, and Associated Design Principles

Care domain	Originating practice		Originating practice needs	Virtual care partner	Model type	Design principles derived
Primary Medical care	Primary care		Immediate care access, increased calendar time for rural/difficult to hire sites, extended hours, and option for nonlocal patients lacking permanent residence	Virtual primary care practice	Collaborative	Immediate access, continuity, convenience, cost, and reach
	Executive primary care		Immediate and 24/7 care access, access for those in occupations or needs that experience frequent travel	Virtual primary care practice	Referral	Immediate access, continuity, convenience
	Remote employees and dependents		Access for nonlocal employees and dependents (eg, children away at college, remote or traveling employees)	Virtual primary care practice	Collaborative	Immediate access, continuity, convenience, and cost
Specialty Medical care	Nephrology	Automated hypertension management program for patients transitioning to primary care/care local to home	Virtual hypertension management practice	Collaborative		Continuity, cost, and transitions
	Rheumatology	Rheumatology medical home for patients lacking rheumatologists local to home, patients in maintenance phase of chronic condition who may need expedited access to tertiary level care	Virtual rheumatology practice	Collaborative & referral		Immediate access, continuity, convenience, cost, reach, and transitions
	Gastroenterology	Personalized digestive care team for patients with stable, chronic digestive disease (eg, inflammatory bowel disease, irritable bowel syndrome)	Virtual gastroenterology practice	Collaborative & referral		Immediate access, continuity, convenience, cost, reach, and transition
	Multiple specialty practices	Medication and behavioral management of patients in need of weight loss to help their comorbid medical condition	Virtual weight management practices	Collaborative & referral		Continuity, convenience, and cost
Mental and behavioral health care	Multiple primary and specialty practices		Medically directed, longitudinal, behavioral support for patients experience chronic pain conditions (eg, fibromyalgia, musculoskeletal pain, chronic headache/migraine, chronic pelvic pain)	Virtual pain reprocessing program	Collaborative & referral	Continuity, convenience, cost, reach, and transitions
	Primary care		Access to virtual, racially, and culturally diverse psychotherapy practice for patient preference, immediate care access, extended hours	Virtual psychotherapy practice	Collaborative & referral	Continuity, convenience, cost, and reach
	Primary care		Access to medically directed behavioral modification for patients seeking weight loss without medication	Virtual behavioral weight management program	Collaborative & referral	Continuity, convenience, and cost

Collaborative models within primary care settings included extension of primary care services outside of traditional office hours and physical locations, additional acute access for existing patients, and a potential primary medical home for nonlocal patients who could benefit from clinical protocols developed and overseen by our specialists in gastroenterology, endocrinology, and hypertension. Referral models were focused on establishing primary medical homes for nonlocal patients receiving care within our specialty practices.

Several opportunities exist for virtual care providers under collaborative care models across specialty medical practices, including oversight for patients with chronic medical conditions who are on stable treatment plans (eg, immunomodulators for autoimmune disorders) but who would benefit from timely access to subspecialty expertise should their clinical condition change and a bridge to local primary care for patients with chronic medical conditions as they stabilize on new treatment plans (eg, diabetes management following pancreatectomy). A referral pathway could also exist for patients exiting the care of a specialty medical provider to a virtual care provider when local access to specialty care is limited or nonexistent.

Mental and behavioral health virtual care providers were identified for both collaborative and referral partnerships. Virtual care providers that enabled longitudinal relationships with patients for nutritional and lifestyle support for weight loss, pain reprocessing therapy, and psychotherapy were identified as important roles within the current clinical practice environment.

### What Design Principles Facilitate the Integration of Virtual Care Delivery Partners into our Traditional, Outpatient Care Delivery System?

Across the 10 clinical use cases analyzed, we identified 6 core design principles that virtual care providers most effectively supported within the shared clinical care model (Table 2). Identified design principles included: (1) immediate access—the ability to supplement traditional care models with immediate care access, for initial and ongoing care needs with acute episodes; (2) continuity—to have mechanisms to facilitate care continuity across care providers to ensure high-quality care and limit redundancies; (3) convenience—to allow for convenience in care for patients to access clinical care outside of our catchment area; (4) cost—to be supported by traditional payment models or arrangements to minimize the financial burden placed on patients; (5) reach—to allow care teams to be able to reach populations who traditionally struggle with local access to primary or specialty-based medical care; and (6) transitions—to enable transitions between care settings when patients may be settling into new care paradigms.

**Table 2:** Design Principles Derived from Case Synthesis Across Virtual Care Provider Models within an Academic Medical Practice

Design principle	Reported experiences
Immediate access	Models that can support traditional care models through immediate care access, for both initial and ongoing care needs with short-term episodes. Models included after-hours access, 24/7 models, and just-in-time access to supplement fully scheduled clinics within both primary care and specialty care practices.
Continuity	Models include the ability to have documentation flow between electronic medical record systems used by referring providers and virtual care providers, ensuring continuity of care and reinforcing the patient experience of having a single, unified medical home.
Convenience	Models included the ability for patients to access care when outside of our catchment area, and virtual care providers offering expanded appointment availability, including same-day access and appointments within 1 hour, and discretion for local patients seeking mental health services within virtual venues.
Cost	Virtual primary and specialty care providers accept standard public and private insurance plans, supporting consistent co-pay structures and minimizing financial ambiguity for patients.
Reach	Models supporting patients residing in areas with limited access to health care services (eg, rural and urban), and virtual care providers with greater cultural and language concordance than available within our health care system.
Transitions	Virtual care providers serve as an immediate next step for patients transitioning from specialty-based care to local primary care providers or moving between other care paradigms.

## Discussion

Within this study, we observed that virtual care providers may serve in both collaborative and referral care models to help address gaps in access to primary, specialty, and behavioral health care services. These findings should be interpreted as descriptive observations from 10 practice-based cases rather than confirmatory evidence of model effectiveness. Virtual care providers serve unique needs for nonlocal patients traveling to an academic medical center for specialty medical care unavailable closer to home, for patients in need of behavioral health or psychological support that is appropriate for virtual care delivery and serving a support mechanism through transitions of care from an academic medical center back to local care settings. We identified 6 distinct design principles to serve as key facilitators to successful integration of virtual care providers into our traditional models of care delivery: acute care access, continuity of care, convenience, cost and insurance coverage, reach to underserved populations, and supportive transitions to home.

Our findings align with prior research suggesting the potential of virtual care providers to extend access to populations in need and improve continuity for patients challenged with logistical or geographic barriers to care.^18^^,^^19^ Notably, our synthesis describes the role of virtual care providers in both collaborative care and referral models in bridging gaps between specialty and primary care, which has not been described in the literature to date. Like previous research, we observed the role of virtual care providers for psychological support for conditions like anxiety and depression but additionally observed the opportunity for pain reprocessing for chronic pain conditions and nutrition and lifestyle support for patients on medications for complex medical conditions.

Successful virtual care partnerships require strong alignment with organizational culture and values, clear expectations for care quality and communication, structured training for health care professionals to adapt to digital tools, and financial models that align with the strengths and capabilities of each entity. Piloting new models on a small scale allows for evaluation and adjustment before broader implementation, whereas seamless referral and transition processes are essential for patient and care team buy-in. Evaluation of patient outcomes and satisfaction will ensure ongoing quality. Robust integration with electronic health records, attention to data security and privacy, and the development of educational resources for both patients and providers help lower barriers and support sustainable adoption. Existing financial models to support these partnerships include fee-for-service billing for some distant-site clinicians, but the evolving landscape of virtual care will increasingly require innovative payment arrangements, such as value-based contracts, bundled payments, and subscription-based services, to ensure the sustainability and scalability of integrated virtual care partnerships. Ongoing regulatory changes and payer policies will continue to shape the feasibility and adoption of these models, underscoring the need for adaptable and forward-looking reimbursement strategies.

Partnerships with virtual care providers present significant opportunities for tertiary and academic medical centers to expand their reach and ensure ongoing care for patients with serious and complex diseases. However, these collaborations also face notable challenges: reimbursement for virtual care remains a hurdle, often requiring virtual care providers to secure contracts with large payors and to keep referring providers informed of coverage changes. The payment landscape is rapidly evolving, with government payers periodically updating policies and coverage for telehealth and virtual services, which introduces uncertainty and complexity for integrated practice models that incorporate virtual care providers. As new payment arrangements emerge, ongoing adaptation to regulatory changes and payer policies will be essential for sustainability. Timely and secure sharing of clinical information between partners using different electronic health record systems is critical for safe and effective care. Furthermore, market forces can impact the financial viability of virtual care providers, making it essential for policies to encourage their participation and reduce the risk of service discontinuation. Addressing these challenges through strong alignment, clear processes, and supportive infrastructure are key to realizing the full potential of partnerships with virtual care providers.

This study has limitations. First, this was a synthesis performed among practices at a single tertiary academic medical center, and local organization structures, contracting approaches, and referral patterns may have shaped the partnerships observed. However, the partnership models, experienced practice needs, and implementation considerations described may be consistent with those experienced within other tertiary care centers, including those managing high referral volumes, geographically dispersed patient populations, limited capacity for new patients, and hiring challenges within certain medical specialties. Despite these limitations, we believe this is the first cross-case description of the opportunities for virtual care providers to become part of an integrated health care ecosystem.

## Potential Competing Interests

The authors report no competing interests.
